# Multimodal Cardiac Imaging in Aortic Regurgitation: From Pathophysiology to Diagnosis and Treatment Planning

**DOI:** 10.3390/jcm15218521

**Published:** 2026-11-02

**Authors:** Malgorzata Maciorowska, Ana Pastorino, Lin Wang, Sylvia Biso, Billal Tokhi, Nadira Hamid, Amro Alsaid, Xiaoli Ren, Timothy Carter, Abdullah Masood, Abdellaziz Dahou, Omar K. Khalique

**Affiliations:** 1 Department of Cardiology and Internal Diseases, Military Institute of Medicine—National Research Institute, Szaserow Street 128, 04-141 Warsaw, Poland; 2 Division of Cardiovascular Imaging, St. Francis Hospital and Catholic Health, Roslyn, NY 11576, USA; anapastorino92@gmail.com (A.P.); omark31@gmail.com (O.K.K.); 3 Minneapolis Heart Institute, Minneapolis, MN 55407, USA; 4 Baylor Scott & White, The Heart Hospital, Plano, TX 75039, USA; 5 Department of Cardiothoracic Surgery, St. Francis Hospital and Catholic Health, Roslyn, NY 11576, USA; 6 Ameer-ud-Din Medical College, Lahore 54000, Pakistan; 7 Geisinger Medical Center, Wilkes-Barre, PA 18711, USA

**Keywords:** aortic regurgitation, echocardiography, cardiac magnetic resonance, cardiac computed tomography, multimodal imaging

## Abstract

Aortic regurgitation (AR) is a complex and often misdiagnosed valvular heart disease. It is often asymptomatic for years, making early detection critical. Once symptoms develop, left ventricular dysfunction may already be irreversible. Timely and accurate grading of AR severity can guide clinical decision-making and improve survival rates. Delayed diagnosis increases the risk of heart failure, arrhythmias, and sudden cardiac death. Transthoracic echocardiography (TTE) is the first-line imaging modality, providing assessment of valve morphology, AR severity, left ventricular size and function, and aortic dimensions. When TTE is inconclusive or additional anatomical information is required, complementary imaging modalities provide incremental value. Transesophageal echocardiography often helps to define AR mechanism and is particularly relevant for valve repair assessment and procedural guidance. Cardiovascular magnetic resonance enables accurate quantification of regurgitant volume and fraction, detailed evaluation of ventricular remodeling, and detection of myocardial fibrosis. Cardiac computed tomography offers high-resolution characterization of the aorta and is increasingly important for surgical and transcatheter planning. In selected patients, multimodality imaging also helps evaluate prosthetic valve dysfunction and suspected infective endocarditis. This paper reviews the currently used imaging modalities and their clinical applicability, and proposes an integrated multimodality imaging approach to guide patient management in chronic AR.

## 1. Introduction

Aortic regurgitation (AR) occurs when the aortic valve fails to close properly, allowing blood to flow backward into the left ventricle (LV) during diastole. It is a complex and often misdiagnosed valvular heart disease [1,2]. Among all valvular lesions, it is the third most common [2,3,4]. The prevalence of moderate or severe AR increases with age, reaching 1.6% in a population over 65 years [2]. The regurgitation is more common in men than in women, probably due to higher prevalence of bicuspid aortic valve, aortopathy, and endocarditis in the former group [3]. Women, however, often present later with more advanced disease and are less likely to be referred to interventional treatment [1,3,5,6]. Delayed diagnosis increases the risk of heart failure (HF), arrhythmias, and sudden cardiac death. Compared to no or mild regurgitation, the risk of mortality is 26% higher in patients with moderate AR and more than double in those with severe AR [7]. Delayed treatment increases the need for emergency surgeries and is associated with worse outcomes. Early intervention reduces long-term morbidity and the cost burden on healthcare systems [8]. AR is asymptomatic in early stages. In advanced disease, dyspnea, fatigue, and palpitation may occur.

**Figure 1:**
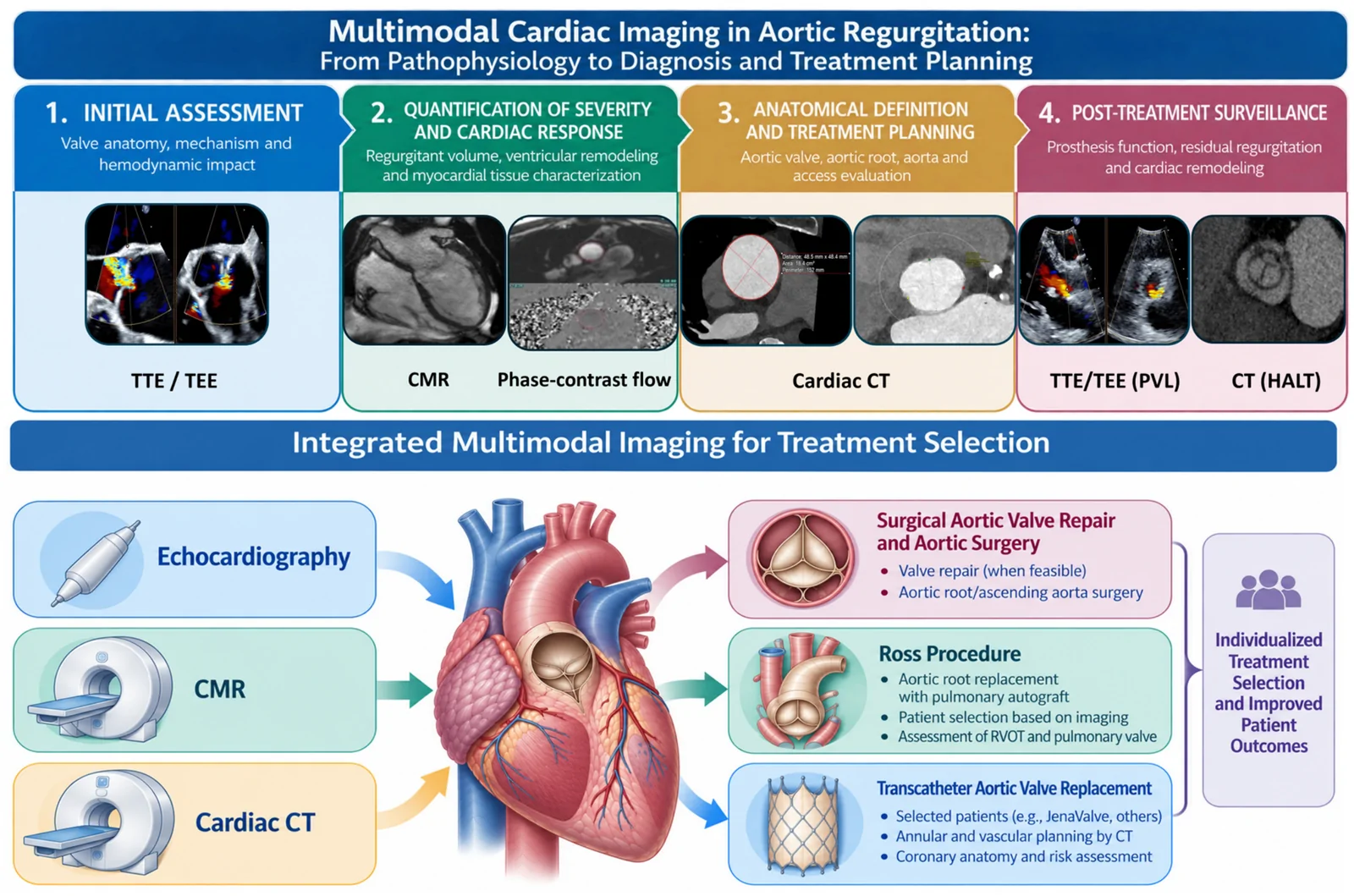
Multimodal cardiac imaging in aortic regurgitation: from diagnosis to treatment selection. Multimodal cardiac imaging provides complementary information throughout the evaluation and management of aortic regurgitation. Echocardiography is the first-line modality for assessment of valve anatomy, mechanism, hemodynamic consequences, and regurgitation severity. CMR provides quantitative assessment of regurgitation and ventricular remodeling, while cardiac CT provides detailed characterization of aortic valve and root anatomy and supports procedural planning. Integration of these modalities informs individualized treatment selection, including surgical aortic valve repair and aortic surgery, the Ross procedure, and transcatheter aortic valve replacement. Generative AI disclosure: illustrative elements in the lower portion of this figure were generated using ChatGPT (OpenAI), based on author-provided instructions and scientific information. All AI-generated elements were reviewed by the authors to ensure scientific accuracy. Abbreviations: CMR, cardiovascular magnetic resonance; CT, computed tomography; HALT, hypoattenuated leaflet thickening; PVL, paravalvular leak; RVOT, right ventricular outflow tract; TEE, transesophageal echocardiography; TTE, transthoracic echocardiography.

**Figure 2:**
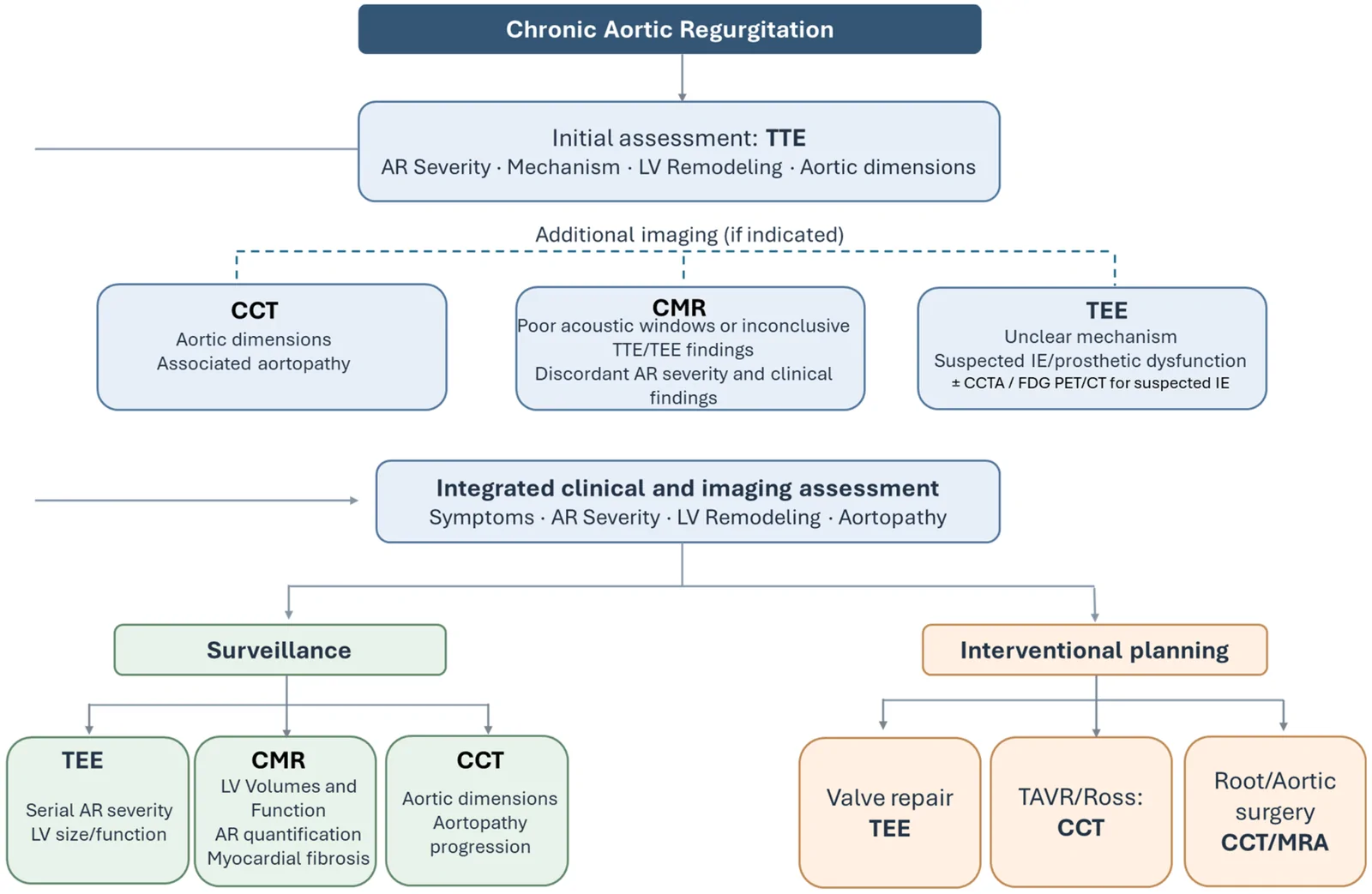
Proposed Multimodality Imaging Algorithm for the Evaluation and Management of Chronic Aortic Regurgitation. TTE is the first-line imaging modality for the evaluation of chronic AR. Additional imaging with TEE, CMR, or CCT is performed selectively according to clinical indications. Integrated clinical and imaging assessment guides longitudinal surveillance and interventional planning. Dashed lines indicate complementary imaging modalities that are not routinely required in all patients. Abbreviations: AR, aortic regurgitation; CCT, cardiac computed tomography; CMR, cardiovascular magnetic resonance; IE, infective endocarditis; LV, left ventricular; MRA, magnetic resonance angiography; Ross, Ross procedure; TAVR, transcatheter aortic valve replacement; TEE, transesophageal echocardiography; TTE, transthoracic echocardiography.

A comprehensive assessment of the severity and mechanism of aortic regurgitation is crucial for accurate diagnosis and selection of an appropriate treatment strategy. Although echocardiography serves as the first-line diagnostic modality, both cardiovascular magnetic resonance (CMR) and cardiac computed tomography (CCT) add valuable information to a thorough evaluation of the disease [9,10]. This paper reviews each of the imaging modalities and their strengths, limitations, and clinical applications and proposes an integrated multimodality approach to the evaluation and management of chronic AR (Figure 1 and Figure 2, and Table 1).

This review was conducted as a narrative rather than a systematic review. No review protocol was registered, and no formal study-selection procedure or risk-of-bias assessment was undertaken. Relevant literature was identified through searches of PubMed and Google Scholar from database inception through October 2026. Studies were selected according to their clinical relevance and the authors’ expertise, prioritizing randomized controlled trials, patient-level meta-analyses, and multicenter studies.

## 2. Anatomy and Mechanisms of Aortic Regurgitation

### 2.1. An Imaging Perspective of the Anatomy of the Aortic Valve

From an imaging perspective, the aortic valve (AV) is a three-dimensional structure within the ventriculo–aortic junction, extending from the left ventricular outflow tract (LVOT) to the proximal ascending aorta. The annulus is a crown-shaped, dynamic structure defined by the hinge line of the cusps, and is typically elliptical rather than circular. The nadirs of the leaflet hinge points define the plane of the “virtual annulus.” The valve comprises three cusps—right coronary (RCC), left coronary (LCC), and non-coronary (NCC)—each attached along a semilunar hinge line and meeting in diastole along the coaptation plane. The RCC is adjacent to the RVOT, and the NCC is adjacent to the interatrial septum. The AV may also develop with one, two (the most common anatomical variant), or four cusps. Above the cusps are the three sinuses of Valsalva, which bulge outward from the aortic root and contain the coronary ostia within the right and left sinuses. The sinotubular junction (STJ), located at the level of the commissures, forms a circumferential ridge that marks the transition to the tubular ascending aorta. Given the valve’s anatomy, comprehensive imaging assessment requires systematic evaluation of each component, ideally using multiplanar and three-dimensional techniques to capture the complex geometry of the aortic root [10,11,12].

### 2.2. Etiology and Mechanism of Aortic Regurgitation

AR may result from either valve-related lesions, aortic root causes, or a combination of both. Intrinsic valvular lesions include leaflet calcifications, bicuspid aortic valve (BAV), infective endocarditis, and rheumatic disease. Functional AR arises from aortic root or ascending aorta dilation, as observed in aortic aneurysm, connective tissue disorders such as Marfan syndrome, and hypertension-induced dilation. Acute AR most commonly results from infective endocarditis, type A aortic dissection, or traumatic injury to the aortic valve or root structures. In contrast, chronic AR is associated with degenerative pathology or BAV accompanied by varying degrees of aortic dilation in more than half of cases [13]. Analogous to Carpentier’s functional classification for mitral regurgitation, AR can be categorized into three types according to cusp motion. Type I indicates normal cusp motion with aortic dilation or cusp perforation, with four subtypes: (a) dilation of the STJ and ascending aorta, (b) dilation of the sinuses of Valsalva and STJ, (c) dilation of the annulus, and (d) cusp perforation. Type II indicates cusp prolapse, and Type III cusp restriction [10,14]. Identification of these mechanisms helps to determine the feasibility of surgical valve repair. Type III predominantly requires aortic valve replacement (AVR), whereas Type is primarily managed with valve-sparing root replacement (VSARR) or targeted annuloplasty, and Type II is highly amenable to surgical repair [15,16].

### 2.3. Left Ventricular Remodeling and Function

Hemodynamics and cardiac adaptation differ substantially between acute and chronic AR. In severe acute AR, the LV is typically nondilated and noncompliant. In contrast, chronic AR is characterized by progressive volume overload leading to LV eccentric hypertrophy and increased total stroke volume as a compensatory response. During the compensated phase, LV dilation and enhanced preload maintain forward cardiac output. With disease progression, continued remodeling results in rising wall stress, declining contractile reserve, LV systolic dysfunction, and ultimately heart failure. Once advanced myocardial remodeling occurs, postoperative recovery may be incomplete. Given the above, accurate quantification of LV size and function is crucial, as it is one of the major determinants of the timing of intervention.

## 3. Echocardiography

Transthoracic echocardiography (TTE) is the first-line imaging modality for the diagnosis and comprehensive assessment of AR, including evaluation of etiology, mechanism, severity, and left ventricular (LV) response. Standard parasternal and apical views should be obtained. AV anatomy and leaflet morphology are best visualized in the parasternal short-axis view (SAX). However, in the presence of significant calcifications, differentiation between a BAV and a tricuspid valve may be challenging. When the image quality permits, three-dimensional (3D) en face visualization improves delineation of valve morphology [17]. The aortic root and proximal ascending aorta are assessed in the parasternal long-axis view, with measurements performed at end-diastole using the leading-to-leading edge method, which correlates best with CCT [18].

Although TTE is often sufficient for grading AR severity, transesophageal echocardiography (TEE) is frequently required to better define the mechanism of regurgitation, to evaluate eccentric jets, or when transthoracic image quality is suboptimal. Standard TEE imaging of the AV includes mid-esophageal short-axis (40–60°) and long-axis (110–140°) using omniplane rotation or biplane imaging. 3D TEE from upper esophageal views provides superior spatial resolution for detailed valve assessment. Transgastric (TG) (0–30° and 110–120°) and deep TG views are useful for evaluating valve function and Doppler interrogation [12]. Diastolic flow reversal in the descending aorta should be assessed from mid- to high esophageal views (0° and 80–100°).

**Figure 3:**
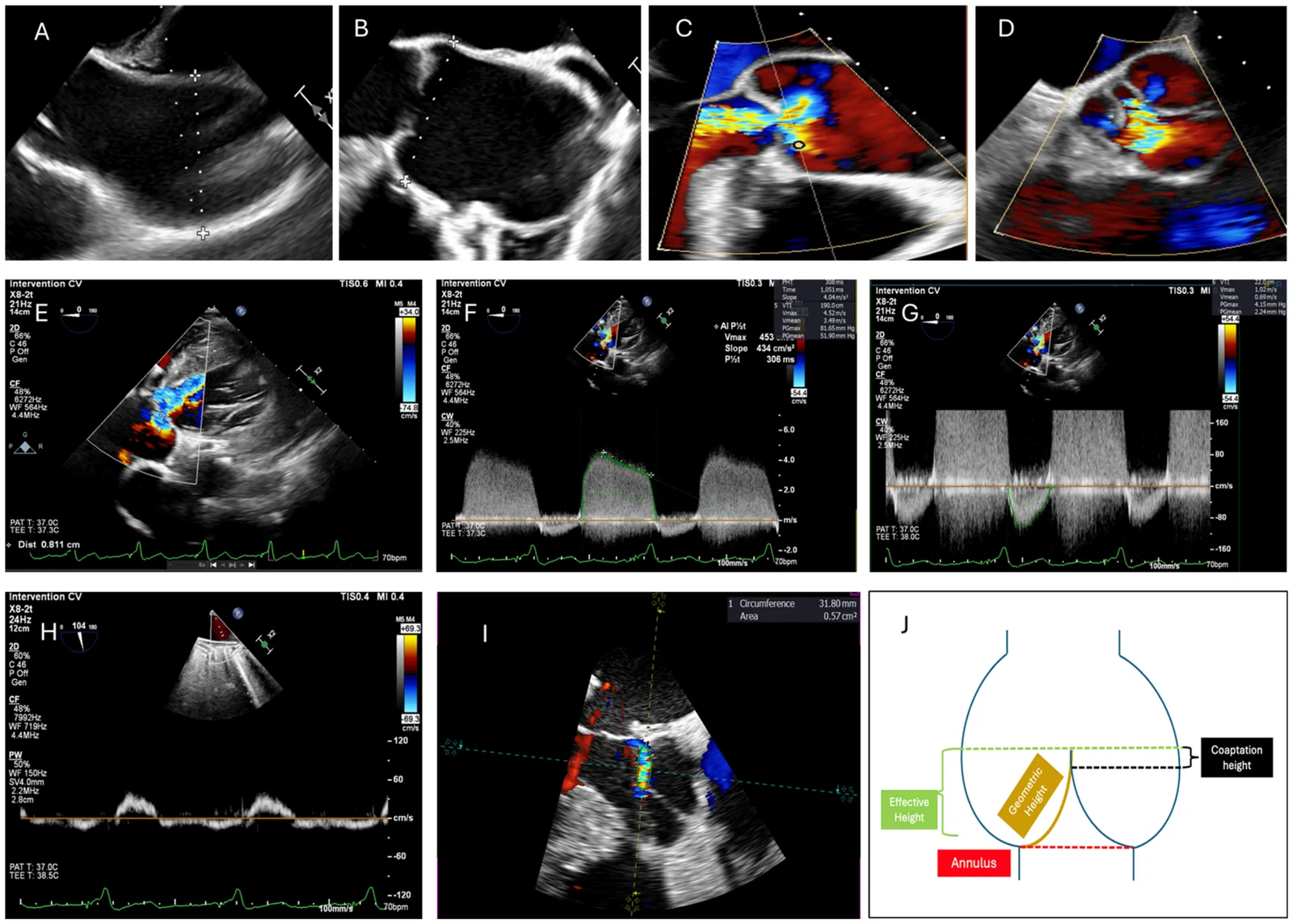
Transesophageal echocardiographic evaluation of severe aortic regurgitation and pre-procedure assessment of aortic valve repair. (**A**–**D**) Type Ia severe aortic regurgitation. (**A**) Dilation of the ascending aorta to 4.9 cm. (**B**) Mild dilatation of the aortic root to 4.0 cm. (**C**,**D**) 2D Biplane Color imaging showing a wide regurgitant jet. (**E**–**H**) AR quantification before TAVI. EROA and regurgitant volume are calculated by PISA ERO, with 0.31 cm^2^, RVol: 67 mL, RF: 73% indicating severe AR. (**H**) Holodiastolic flow reversal in the descending aorta. (**I**) 3D Multiplanar reconstruction with Color Doppler, showing VCA 3D measuring 0.57 cm^2^. (**J**) Echocardiographic measurements of the aortic root performed during intraprocedural assessment for aortic valve repair. Abbreviations: AR, aortic regurgitation; EROA, effective regurgitant orifice area; RF, regurgitant fraction; RVol, regurgitant volume, VCA, vena contracta area.

Current guidelines recommend a multiparametric approach integrating qualitative, semi-quantitative, and quantitative parameters for accurate AR assessment [9,10,13]. Of note, no single parameter should be interpreted in isolation, particularly when parameters are discordant. Despite established recommendations, echocardiographic quantification of AR remains challenging in routine clinical practice [19] (Figure 3A–E).

### 3.1. Assessment of AR Severity

#### 3.1.1. Qualitative Parameters

Qualitative assessment includes AV morphology, color Doppler jet appearance, continuous-wave (CW) Doppler density and contour, and diastolic flow reversal in the descending and abdominal aorta [9,10,13]. A faint or incomplete CW envelope corresponds to mild or trace regurgitation, whereas a dense envelope indicates more severe regurgitation, but does not reliably distinguish moderate from severe AR. Notably, eccentric jets may appear disproportionately large due to the Coanda effect; therefore, jet length or jet area alone from any window should not be used for grading severity. Holodiastolic flow reversal in the descending aorta, assessed with pulsed-wave Doppler (PW) just distal to the subclavian artery, with end-diastolic velocity exceeding 20 cm/s, is a simple supportive marker of severe AR and is more specific when present in the abdominal aorta (Figure 3H). The parameter has a strong correlation with CMR regurgitant volume (RVol) and regurgitant fraction (RF) [20]. Since it depends on compliance of the aorta, it is less reliable in older patients. In milder degrees of AR, a reversal is limited to early diastole. However, a brief early diastolic reversal is often observed in normal studies.

#### 3.1.2. Semi-Quantitative Parameters

Semi-quantitative markers include vena contracta (VC) width, pressure half-time (PHT), color jet width and its ratio to the LVOT diameter, and jet area and its ratio to LVOT area [9,10,13].

The VC represents the narrowest portion of the regurgitant jet immediately below the convergence region and is a surrogate for the size of the regurgitant orifice. With a Nyquist limit of 50–60 cm/s, a VC less than 3 mm suggests mild AR, whereas a value of >6 mm indicates severe regurgitation. Intermediate values need confirmation by more quantitative methods. The VC can be used in eccentric jets. Widths of multiple jets are not additive. Compared with CMR, 2D VC width is one of the most reliable single echocardiographic discriminators of severe AR [21]. The VC concept assumes a circular regurgitant orifice; however, it is often irregular or elliptic, resulting in inter-view variability. Measurements may be overestimated in TG views due to suboptimal lateral or azimuthal resolution. 3D echo enables direct planimetry of the VC area, improving geometric accuracy (Figure 3I). Current data support a 3D VCA > 26 mm^2^ as a CMR-validated threshold for severe AR, while ≥30 mm^2^ is a cut-off for identifying asymptomatic patients at higher risk who may need earlier valve surgery [20,22].

PHT, derived from CW, reflects the rate of equalization of the diastolic pressure gradient between the aorta and LV. In practice, very short values (<200 ms) are used as a supportive marker of severe AR; intermediate values are unreliable for grading. Misalignment of the CW Doppler cursor, eccentric or multiple jets affect the accuracy of measurements. Measurements are also influenced by LV compliance, systemic vascular resistance (including vasodilator therapy), and LV systolic dysfunction. Among echo parameters, PHT had the lowest accuracy when compared to CMR [23].

Jet width/LVOT diameter ratio is a color Doppler parameter measured in the PLAX view that compares the jet width at its origin to the LVOT diameter. It provides a proportional estimate of AR severity but is influenced by technical settings and jet eccentricity, limiting standalone reliability. A numerical value less than 25% is consistent with mild AR, and a value of ≥65% indicates severe AR.

The jet area/LVOT area ratio is based on planimetry of the color Doppler jet and its proportion to LVOT area. These measures are highly load- and gain-dependent and may overestimate severity in eccentric jets (Coanda effect), so they should not be used in isolation for grading AR. A value of ≥60% indicates severe AR [9,10].

#### 3.1.3. Quantitative Parameters

Quantitative assessment using the proximal isovelocity surface area (PISA) method or volumetric Doppler techniques provides effective regurgitant orifice area (EROA), RVol, and RF (Figure 3E–G). An EROA ≥ 30 mm^2^ or an RVol ≥ 60 mL/beat indicates severe AR. An RF ≥ 50% is also consistent with severe regurgitation and may indicate hemodynamically significant AR when EROA and RVol fall within the moderate range [9]. Current guidelines recommend the use of the PISA method whenever feasible, although it is technically demanding and requires experience [9]. It is most reliable when regurgitant orifice geometry is favorable and the flow convergence zone is clearly visualized. Because the radius measurement is squared in the calculation, small measurement errors result in substantial misestimation of EROA. In eccentric jets, the PLAX view may better delineate the flow convergence region. Feasibility is limited by AV calcifications, poor acoustic windows, or a non-circular regurgitant orifice, commonly seen in BAV. The PISA method is not valid in the presence of multiple jets. Volumetric methods, based on the continuity principle, can be applied to multiple and eccentric jets. However, they are operator-dependent. Table 2 summarizes multimodality imaging parameters and reported cut-offs for severe AR across echocardiography, CMR, and CCT.

### 3.2. Left Ventricular Remodeling and Function Assessment by Echocardiography

Preoperative LVEF and left ventricular end-systolic diameter indexed (LVESDi) to body surface area (BSA) were found to be predictive of reverse remodeling 12 months after surgery [26]. In a retrospective cohort study, a left ventricular end-systolic volume index (LVESVi) threshold ≥ 45 mL/m^2^ was significantly associated with an increased mortality risk in patients with asymptomatic regurgitation [27]. Recent European guidelines recommend earlier intervention in asymptomatic severe AR with low surgical risk in the presence of significant LV dilation (left ventricular end-systolic diameter (LVESD) > 50 mm, or LVESDi > 25 mm/m^2^) or of LV systolic dysfunction (LVEF ≤ 50%) (Class I) [13]. They also suggest that earlier surgery might be beneficial for long-term prognosis in patients with LVESDi > 22 mm/m^2^ or LVESVi > 45 mL/m^2^, especially in patients with small body size (BSA < 1.68 m^2^) or resting LVEF ≤ 55% (Class IIb). The 2020 ACC/AHA guidelines use the same thresholds for LVESD and LVESDi in asymptomatic patients, but with a lower class of recommendation (Class 2a) and recommend surgery once LVEF falls ≤ 55% (Class I). They consider intervention reasonable if progressive decline in LVEF occurs in the 55–60% range on serial imaging (Class IIb) [28] [Table 3].

Current intervention cut-offs are largely based on 2D echocardiographic measurements. However, 3D echocardiography provides a more accurate assessment of LV volumes and LVEF and has high correlation with CMR [9,29]. Importantly, several studies have demonstrated sex-related differences in LV remodeling in response to chronic AR [30,31,32,33]. Women and older patients tend to exhibit smaller LVEDVi and LVESVi indices and less pronounced dilation as AR severity increases. These differences are not fully explained by body size alone and raise concern for potential underestimation of disease by TTE. This observation underscores the importance of multimodality imaging and may support the implementation of sex-specific thresholds for intervention.

Advances in imaging technology have improved the detection of LV subclinical dysfunction in AR. Global longitudinal strain (GLS) has emerged as an early prognostic marker, identifying subclinical cardiac impairment in asymptomatic patients with moderate-to-severe AR and preserved LVEF, thereby helping to optimize the timing of intervention [34,35]. In patients with severe chronic AR undergoing surgery, a baseline value < 19% was associated with increased postoperative mortality [35]. LA remodeling progresses in parallel with worsening AR, with similar rates across age and sex [36]. LA strain, particularly reservoir strain, is a sensitive marker of LA dysfunction and may add prognostic value beyond LV GLS, LV size, and clinical variables in asymptomatic severe AR [36,37]. An LA reservoir strain ≤35% and an LA volume index ≥ 37 mL/m^2^ were associated with adverse cardiovascular events [36]. Myocardial work, a novel echocardiographic method that integrates LV mechanics with noninvasively estimated afterload, represents a promising tool for predicting post-operative dysfunction in patients with chronic severe AR and preserved LVEF [38].

### 3.3. Transesophageal Intraoperative Assessment for Aortic Valve Repair

Although AV replacement remains the standard surgical approach in most AR cases, VSARR and AV repair are increasingly performed in high-volume centers, with favorable long-term outcomes [13]. TEE plays a role at different stages of AV repair, from preoperative confirmation of the mechanism and assessment of repairability to intraoperative guidance and identification of anatomical predictors of late failure [39,40,41,42,43,44]. Intraoperatively, 2D mid-esophageal short- and long-axis views, together with deep TG 5-chamber views, are used to evaluate the AV. 3D imaging provides more accurate evaluation of cusp geometry and enables more precise height measurements. In a study of 163 consecutive patients, preoperative TEE predicted repairability, with 86% accuracy for repair and 93% for replacement, with excellent agreement between TEE and surgical inspection for classification of AR mechanism (93%; kappa = 0.90) [45].

Beyond standard AV evaluation, preoperative TEE focuses on aortic root dimensions (annulus, sinuses of Valsalva, STJ), cusp tissue quality (the presence of calcifications, fibrosis, fenestrations, or perforation) and motion (retraction, prolapse), valve geometry (cusp geometric and effective height, coaptation level and length), and AR jet direction. Effective height is the orthogonal distance from the virtual basal ring plane to the coaptation plane of the cusps, with a target of approximately 9–10 mm after repair [46]. The most important quantitative preoperative parameter is geometric height (gH), which is a curvilinear distance measured from the cusp nadir to the midpoint of the free margin and reflects the amount of cusp tissue. A preoperative gH > 17 mm for tricuspid valve (TAV) and >20 mm for BAV favors repair, whereas a gH ≤ 16 mm in TAV and ≤19 in BAV indicates cusp retraction and poor repairability [47]. Coaptation length represents the extent of leaflet apposition during diastole, with a post-repair value > 4 mm considered favorable [44]. These measurements provide important information regarding valve repairability and the adequacy of cusp coaptation [40,42,48]. Figure 3J provides a schematic representation of the measurements obtained by TEE. For BAVs, commissural orientation is particularly important; more symmetric valves, typically with a commissural orientation of 160–180°, are associated with better durability. Repair remains feasible in moderately asymmetric valves (commissural orientation of 140–159°), but outcomes are less favorable in very asymmetric valves with orientation < 140° [49].

Following repair, TEE should be performed after complete separation from cardio-pulmonary bypass under physiological loading conditions, with adequate left ventricular filling, blood pressure, and heart rate. TEE is pivotal for confirming adequate cusp coaptation, excluding significant residual regurgitation, and identifying persistent cusp prolapse [42,50]. More than mild residual AR, especially if eccentric, should prompt careful reassessment of the mechanism and may indicate the need for immediate re-repair. Effective height, coaptation height and level, aortic annulus diameter, and mean transvalvular gradient should be reevaluated after repair. Features associated with a favorable and durable repair include: no or trivial residual AR, coaptation above the aortic annulus, an effective height > 9 mm, a coaptation length > 4 mm, adequate annular reduction and stabilization when the annulus is dilated, and a mean post-repair transvalvular aortic gradient not exceeding approximately 10–15 mmHg [39,42,48].

## 4. Cardiovascular Magnetic Resonance

**Figure 4:**
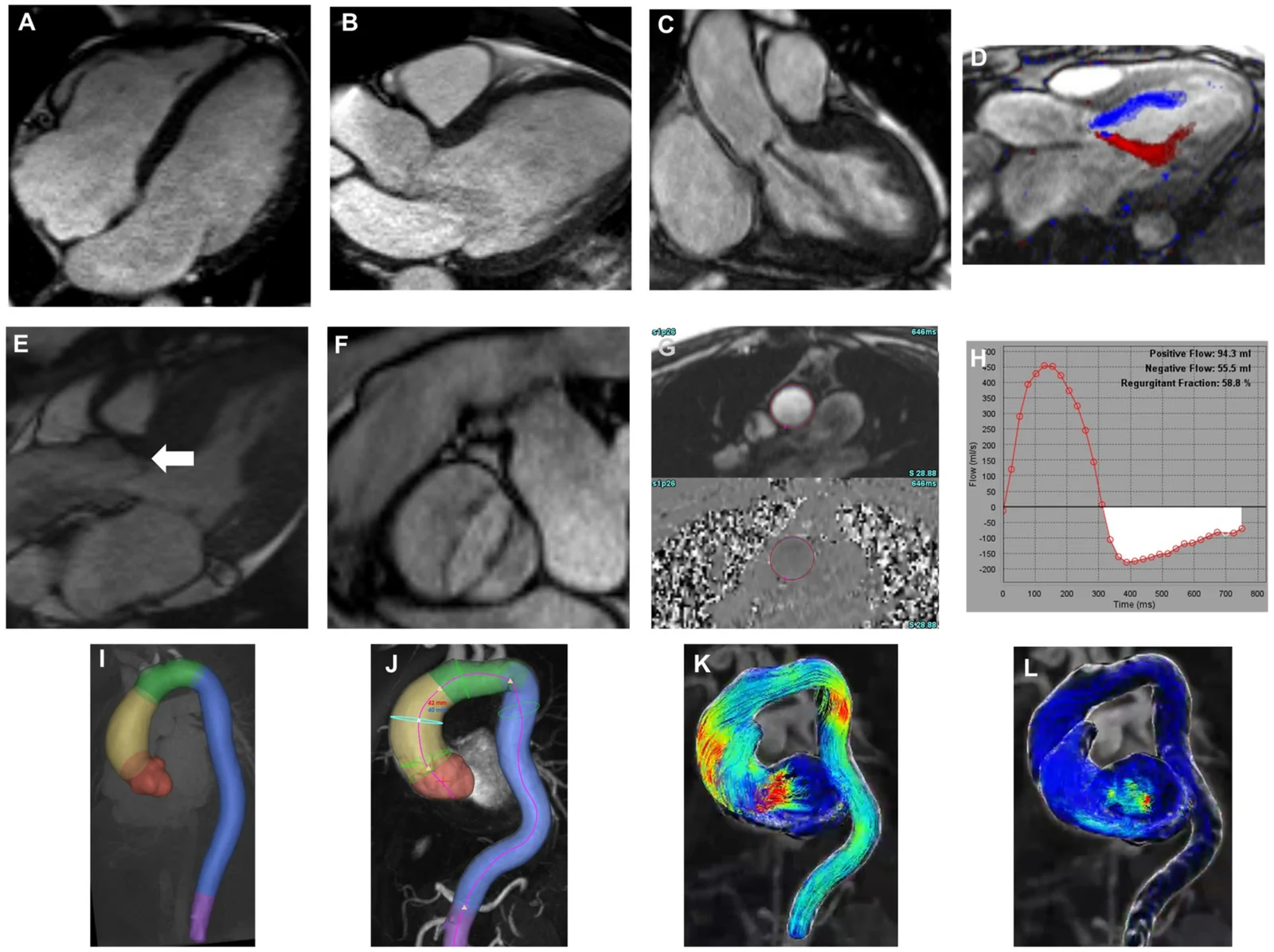
Cardiovascular magnetic resonance assessment of aortic regurgitation and associated aortic pathology. (**A**) Cine CMR, 4-chamber view. Dilated LV. (**B**) Cine CMR, 3-chamber view. Dilated LV with visualization of the LVOT, aortic valve, and proximal ascending aorta. (**C**) Cine CMR, LVOT view. Diastolic aortic regurgitant jet extending from the aortic valve into the LVOT. (**D**) Color-coded aortic regurgitant jet. Color overlay demonstrating the direction and spatial distribution of regurgitant flow within the LVOT. (**E**) Cine CMR, 3-chamber view. Subaortic membrane within the LVOT (white arrow). (**F**) Cine CMR, aortic valve short-axis view. Bicuspid aortic valve morphology. (**G**) Two-dimensional phase-contrast imaging of the ascending aorta. Magnitude and phase images with a region of interest positioned within the aortic lumen for quantitative flow assessment. (**H**) Two-dimensional phase-contrast aortic flow quantification. Time–flow curve demonstrating antegrade systolic and retrograde diastolic flow, with a regurgitant fraction of 59%, consistent with severe AR. (**I**) Three-dimensional segmentation of the thoracic aorta. Color-coded reconstruction of thoracic aortic anatomy. (**J**) Three-dimensional segmentation and centerline analysis of the thoracic aorta. Multiplanar reconstruction along the aortic centerline for systematic assessment of aortic dimensions, demonstrating ascending aortic dilatation. (**K**) Four-dimensional flow CMR velocity map during systole. Systolic flow through the aortic root and ascending aorta and its spatial distribution within the thoracic aorta. (**L**) Four-dimensional flow CMR velocity map during diastole. Retrograde diastolic flow associated with moderate AR (RF, 42%). Abbreviations: AR, aortic regurgitation; CMR, cardiovascular magnetic resonance; LV, left ventricle; LVOT, left ventricular outflow tract; RF, regurgitant fraction.

CMR provides powerful analysis for the assessment of AR and its associated consequences. CMR holds a class 1 recommendation for the evaluation of AR when echocardiographic assessment is equivocal in the 2020 ACC/AHA Guidelines on Valvular Disease [28]. The 2025 European Society of Cardiology (ESC) guidelines position CMR as the superior modality for measuring prognostic diagnostic LV dilation in the setting of chronic AR [13]. Cine CMR provides complementary assessment of LV remodeling, aortic valve and LVOT morphology, and associated structural abnormalities (Figure 4A–F). AR is quantified by two-dimensional (2D) phase-contrast (PC) imaging, which is a routine application of CMR. Using a region of interest at the plane of the STJ, direct measurements of the forward stroke volume and RVol can be performed, and RF can also be calculated (Figure 4G–H). 2D PC measurements by CMR exhibit superior reproducibility to echocardiographic assessment of AR [51].

### 4.1. CMR Quantification of AR: Diagnostic and Prognostic Implications

#### 4.1.1. CMR-Derived Regurgitant Fraction and Volume: Diagnostic and Prognostic Thresholds

The 2025 Society for Cardiovascular Magnetic Resonance (SCMR) reference values update presents thresholds for severe AR (RVol ≥ 60 mL and RF ≥ 50%), which, as stated by the authors, were adapted from echocardiographic criteria [25]. This highlights the lack of established, CMR-specific diagnostic thresholds for grading AR severity. Nevertheless, several observational studies have identified CMR-derived RF and RVol thresholds associated with clinical outcomes or progression to AVR [Table 4].

#### 4.1.2. Differences Between Echocardiographic and CMR-Derived Thresholds

The apparently lower CMR-derived thresholds reported in observational studies compared with conventional echocardiographic criteria for severe AR should be interpreted in context. First, these thresholds are not directly comparable, as they were established for different purposes: echocardiographic cutoffs are used to define AR severity, whereas the lower CMR-derived thresholds were identified based on their association with clinical outcomes. Echocardiographic cutoffs for diagnostic severity have been historically used and are very specific. However, the original validation leading to the severe cutoffs was not based on prognostic data but rather visual interpretation [54]. Second, echocardiography and CMR rely on different methodologies for AR quantification, and their measurements and corresponding thresholds should not be considered interchangeable. In a study of 81 patients with chronic AR (58% with BAV), Attar et al. reported only moderate agreement in AR severity classification between echocardiography and CMR (64.2%; κ = 0.48), despite applying the same severity thresholds. Although regurgitant volume measurements correlated well (r = 0.79), Bland–Altman analysis revealed wide 95% limits of agreement (−35.9 to +36.0 mL), highlighting substantial variability in individual measurements between modalities [21]. Further prognostic studies are needed to establish and validate CMR-derived thresholds for AR severity and risk stratification.

CMR-derived RF and RVol are related but not interchangeable parameters. While RVol represents the absolute volume of regurgitant flow, RF expresses this volume relative to the total forward flow and is influenced by the magnitude of forward SV. Consequently, these parameters may yield different severity classifications even within the same modality. In the study by Attar et al., RF and RVol-based CMR severity grades were discordant in 27% of patients, with RVol indicating a higher severity grade in 21 of these 22 cases [21]. Nevertheless, both parameters have demonstrated prognostic value. In the study by Myerson et al. summarized in Table 4, RF and RVol predicted progression to symptoms or conventional surgical indications, with AUCs of 0.93 and 0.96, respectively [52].

#### 4.1.3. Managing Echocardiography–CMR Discordance: Toward an Integrated Assessment of Aortic Regurgitation

Both echocardiography and CMR have inherent limitations that should be considered when interpreting discordant findings. Echocardiographic parameters vary in feasibility and diagnostic performance, particularly in the presence of eccentric regurgitant jets. In the aforementioned study by Attar et al., volumetric quantification using the difference between LVOT and mitral annular SV showed the highest feasibility (98.8%), whereas PISA was feasible in only 37% of patients. Among nonvolumetric parameters, VC width showed the highest diagnostic accuracy for identifying severe AR by CMR (AUC = 0.93; cutoff ≥ 0.64 cm), while LVEDVi also demonstrated good discriminative performance (AUC = 0.84; cutoff ≥ 93 mL/m^2^) [23]. CMR phase-contrast quantification can be affected by plane positioning, complex or eccentric flow, and background phase-offset errors, as discussed in Section 4.3.1.

**Figure 5:**
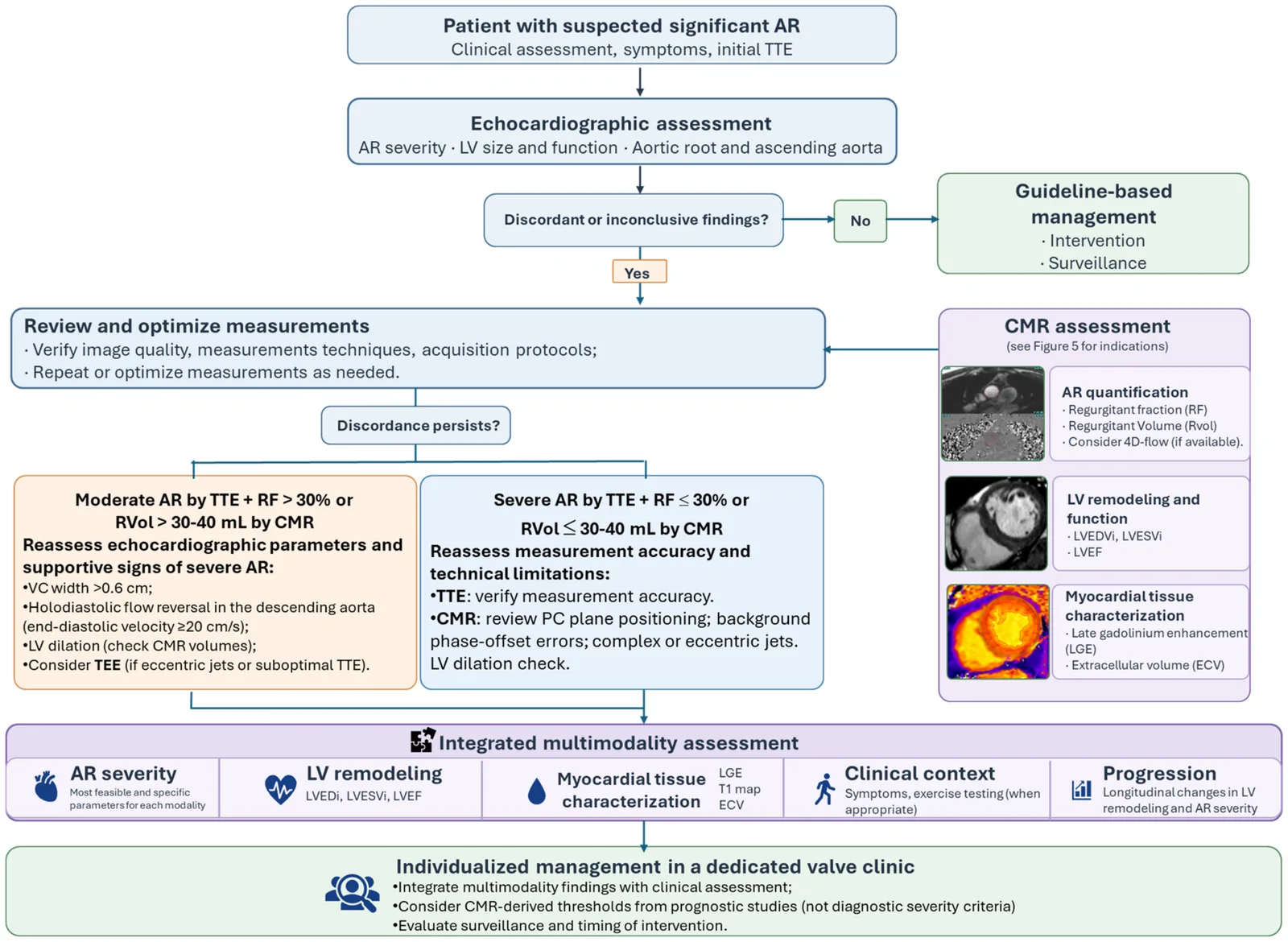
Proposed diagnostic algorithm for the integrated assessment of echocardiography–CMR discordance in AR. The algorithm outlines an approach to patients with suspected significant AR and discordant or inconclusive findings between ETT and CMR. It emphasizes reassessment of measurement accuracy and technical limitations, integration of AR quantification with LV remodeling and myocardial tissue characterization, and consideration of clinical findings to guide individualized management. CMR-derived prognostic thresholds should not be interpreted as interchangeable with echocardiographic criteria for AR severity. Abbreviations: AR, aortic regurgitation; CMR, cardiovascular magnetic resonance; ECV, extracellular volume; LGE, late gadolinium enhancement; LV, left ventricular; RF, regurgitant fraction; RVol, regurgitant volume; TEE, transesophageal echocardiography; TTE, transthoracic echocardiography.

When echocardiography and CMR yield discordant severity assessments, we propose a stepwise diagnostic approach (Figure 5). The first step is to verify image quality, acquisition protocols, and measurement accuracy, repeating or optimizing measurements as needed. If discordance persists, an integrated assessment should prioritize the most feasible and specific parameters from each modality, considering local expertise and technical availability. In patients with moderate AR by echocardiography (ASE criteria: RVol 30–59 mL, RF 30–49%, or EROA 0.10–0.29 cm^2^) but elevated CMR-derived RF or RVol (>30% or >30–40 mL, respectively), more specific echocardiographic markers of severe AR should be reassessed, including VC width (>0.6 cm) and holodiastolic flow reversal in the descending aorta (end-diastolic velocity ≥ 20 cm/s), along with supportive evidence of LV remodeling, particularly LV dilation [10]. Conversely, when echocardiography suggests severe AR (RVol ≥ 60 mL, RF ≥ 50%, or EROA ≥ 0.30 cm^2^) but CMR-derived RF is ≤30% or RVol is ≤30–40 mL, potential technical limitations of both modalities should be reassessed. CMR-derived thresholds have been reported as describing “significant” AR based on prognostic studies rather than as diagnostic severity cutoffs [56].

In cases of persistent discordance, clinical decision-making should integrate LV volumes and LVEF, symptoms, exercise testing when appropriate, and serial changes in LV remodeling and AR severity [13]. CMR tissue characterization may provide complementary information, although its role in guiding intervention remains incompletely validated [13]. Patients with significant AR, particularly those with discordant imaging findings, should preferably be followed in a dedicated valve clinic to support individualized surveillance and timely intervention when indicated.

### 4.2. Evaluation of Left Ventricular Remodeling and Myocardial Fibrosis

#### 4.2.1. Left Ventricular Volumetric Assessment and Remodeling

**Figure 6:**
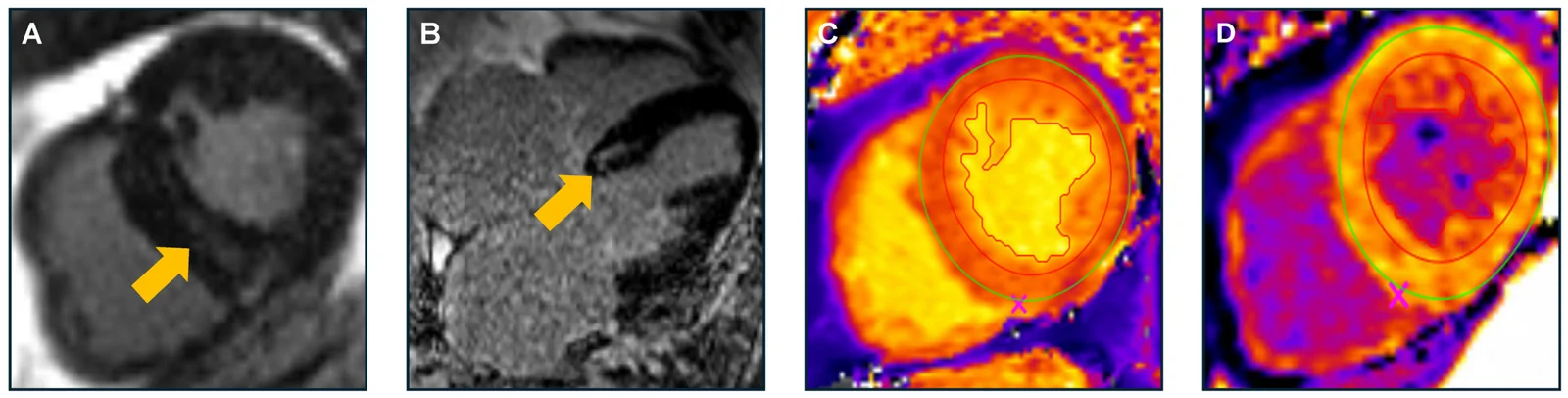
CMR assessment of myocardial fibrosis in a patient with severe AR. (**A**) LGE in a mid-ventricular short-axis view demonstrating mid-wall enhancement in the anteroseptal and inferoseptal segments (yellow arrow). (**B**) Four-chamber view demonstrating LGE mid-wall enhancement in the basal inferoseptal segment (yellow arrow). In panels (**C**,**D**), the contours delineate the myocardium, while the innermost contour defines the left ventricular blood pool. The magenta cross indicates the right ventricular insertion point, automatically identified by the post-processing software and manually adjustable if necessary. (**C**) Native T1 mapping in a basal short-axis view demonstrating increased myocardial native T1 (1064 ms) at 1.5 T, suggestive of diffuse interstitial fibrosis. (**D**) Post-contrast T1 mapping used for ECV quantification, with a resulting ECV of 33%, suggestive of diffuse interstitial fibrosis. Abbreviations: AR, aortic regurgitation; CMR, cardiovascular magnetic resonance; ECV, extracellular volume; LGE, late gadolinium enhancement.

CMR is the gold standard for volumetric assessment of chamber volumes and visualization of LV fibrosis [Figure 4A,B and Figure 6A,B]. LV volumetric analysis is performed by Simpson’s method at end-diastole and end-systole to determine LVEF [57]. Normative data demonstrate differences between men and women, even when indexed [25,58]. LV volumetric assessment by CMR is highly accurate, with experimental ex vivo animal heart models demonstrating excellent correlation with known ventricular volumes (r > 0.99) [59]. Reproducibility is also excellent in patients with chronic AR, with interobserver intraclass correlation coefficients of 0.998 and 0.997 for LVEDV and LVESV, respectively [53].

Assessment of LV volumes indexed to BSA is particularly relevant in chronic AR, as it may influence the timing of intervention. The 2025 ESC/EACTS guidelines include an LVESVi threshold > 45 mL/m^2^ among the criteria for which surgery may be considered in asymptomatic patients with severe AR and low surgical risk (Class IIb, Level B) [13]. Although the formal recommendation cites evidence from both echocardiographic and CMR studies [27,53,55,60,61,62,63], the accompanying text distinguishes the echocardiography-based threshold of >45 mL/m^2^ from a recently proposed CMR-derived threshold of ≥43 mL/m^2^ [55]. In the context of known underestimations of LV volumes by echocardiography as compared to CMR, the CMR-derived threshold is likely met at a lower level of LV dilatation [23]. Given that LV volumes obtained by 2D echocardiography, 3D echocardiography and CMR are not directly interchangeable, modality-specific thresholds should be considered when interpreting these findings.

In addition to imaging modality, sex-specific differences should also be considered when assessing LV remodeling. Lopez Santi et al. identified sex-specific echocardiographic LVESVi thresholds associated with mortality in chronic AR (≥40 mL/m^2^ in women and ≥45 mL/m^2^ in men), highlighting the need for further studies to establish sex-specific CMR-derived thresholds [64].

Furthermore, Vejpongsa et al. demonstrated that a lower CMR-derived RF was associated with a similar degree of LV dilatation in AR compared with MR (40% vs. 50% for an LVEDVi of 100 mL/m^2^), highlighting lesion-specific differences in ventricular remodeling [54].

#### 4.2.2. Myocardial Tissue Characterization and Fibrosis

Native T1 mapping enables quantitative assessment of myocardial longitudinal relaxation times without gadolinium administration, providing a sensitive, although nonspecific, marker of diffuse myocardial tissue alterations. Elevated native T1 values may reflect interstitial fibrosis or edema, potentially identifying subclinical myocardial maladaptation not detectable by late gadolinium enhancement (LGE). In chronic AR, native T1 mapping may complement extracellular volume (ECV) quantification in characterizing myocardial remodeling associated with volume overload (Figure 6C,D). Unlike native T1 mapping, ECV quantification requires pre- and post-contrast T1 measurements and hematocrit correction, providing an estimate of myocardial extracellular expansion [65]. In a cohort of 177 patients with isolated chronic AR undergoing CMR, Senapati et al. evaluated the prognostic value of replacement and diffuse interstitial fibrosis over a median follow-up of 2.5 years. Indexed ECV increased with AR severity (*p* < 0.001). iECV remained independently associated with the composite outcome of death or AVR (*p* = 0.01), with the highest event rates observed in patients with AR regurgitant fraction ≥ 30% and iECV ≥ 24 mL/m^2^ [66].

Late gadolinium enhancement (LGE) enables the detection of focal myocardial replacement fibrosis, which has been associated with adverse clinical outcomes across various cardiovascular diseases [67]. In the setting of AR, LGE may appear in a mid-wall pattern (non-ischemic fibrosis) (Figure 6A,B) [68]. Similarly to the concepts presented for aortic stenosis, Malahfji et al. suggested a staging system based on progressive upstream effects of AR, including CMR LV fibrosis, mitral valve/left atrial disease, and right heart remodeling [69].

### 4.3. Limitations and Emerging CMR Techniques

#### 4.3.1. Technical Limitations of 2D Phase Contrast

The accuracy of 2D phase-contrast CMR for AR quantification is influenced by measurement plane selection, particularly in patients with complex flow patterns, such as those with BAVs, a common etiology of AR. In a study by Muzzarelli et al., forward flow measured in the ascending aorta was underestimated by 22% relative to LV stroke volume in patients with BAVs, compared with 9% in controls. Flow measurements performed at the level of the AV and left ventricular outflow tract (LVOT) showed better agreement with LV stroke volume [70]. These findings may have implications for the calculation of RF in patients with AR. Current standardized SCMR protocols recommend positioning the acquisition plane approximately 5 mm above the aortic valve at end-diastole for AR quantification [57].

Inappropriate velocity encoding (VENC) may introduce additional errors in 2D phase-contrast CMR, resulting in velocity aliasing when set too low or reduced velocity-to-noise ratio when set too high. Background phase offset errors may also introduce systematic inaccuracies in flow quantification, potentially affecting RVol and RF measurements. Appropriate VENC selection and background phase correction are therefore essential for accurate flow quantification [71].

#### 4.3.2. General Limitations of CMR

CMR has several practical limitations, including limited availability and long examination times. Prolonged acquisitions and repeated breath-holding may be challenging in elderly patients, while arrhythmias may compromise ECG-gated image quality. Myocardial tissue characterization using LGE and ECV requires gadolinium-based contrast administration, warranting caution in patients with acute kidney injury—with newer agents, safety with chronic kidney disease has been established [72].

#### 4.3.3. Emerging Techniques

Modern CMR real-time sequences allow adequate volumetric analysis without the need for breath hold. New AI sequence accelerators may allow multiple slices to be acquired rapidly using segmented techniques [73]. Averaging two to three 2D PC acquisitions allows for adequate assessment of flow in the presence of atrial fibrillation [74].

Time-resolved, 3D phase-contrast imaging (4D flow CMR) is a more advanced tool with a growing body of evidence (Figure 4K–L). The advantages include direct measurement of valvular regurgitation, including for atrioventricular valves, and the ability to align measurements of regurgitant jets using multi-planar reconstruction [75]. Aortic wall-shear stress can also be calculated using a dedicated software package. Wall-shear stress has been associated with regional progressive aortic dilation in BAVs [76] and has the potential to guide therapy in the future. Advances in respiratory-navigated 3D magnetic resonance angiography (MRA), particularly image-based navigation (iNAV), enable free-breathing assessment of the aortic root and ascending aorta with improved respiratory motion correction and measurement reproducibility. Emerging self-navigation techniques may further simplify acquisition, and clinical implementation is expanding. Both MRA and CCT hold class 1 recommendations for measurement of the aorta to assess for presence or progression of aortic disease [77,78] (Figure 4I,J).

## 5. Computed Tomography

Cardiac computed tomography angiography (CCT) has emerged as a valuable complementary imaging modality, particularly when detailed anatomical assessment of the AV and root is required [56].

One of the main advantages of CCT is that it is an inherently 3-dimensional volumetric dataset with the highest spatial resolution of the cardiac imaging modalities.

### 5.1. Assessment of Aortic Valve, Root, and Ascending Aorta

**Figure 7:**
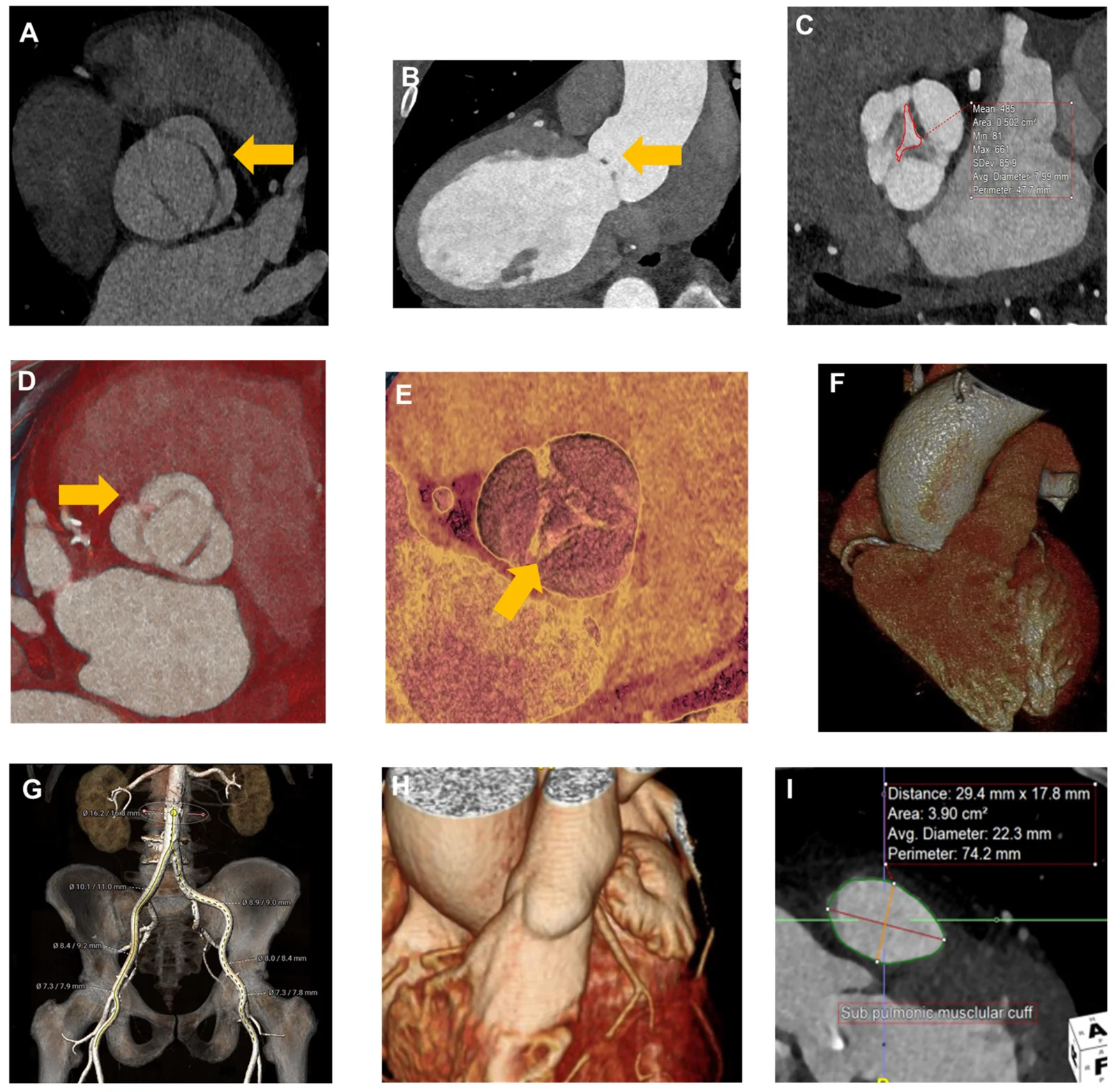
Computed tomography assessment of aortic regurgitation and procedural planning. (**A**) Axial CT plane. Bicuspid aortic valve, Sievers type 1, with a raphe (yellow arrow) between the left coronary cusp (LC) and right coronary cusp (RC). (**B**) Multiplanar CT reconstruction of the LVOT and aortic root. Left ventricular dilation and diastolic malcoaptation of the aortic valve leaflets (yellow arrow). (**C**) Axial CT plane. Direct planimetry of the anatomic aortic regurgitant orifice area (0.5 cm^2^). (**D**) Aortic valve CT reconstruction, leaflet view. Bicuspid aortic valve, Sievers type 1, with a raphe (yellow arrow) between the LC and RC. (**E**) Volume-rendered CT reconstruction of the aortic valve. Diastolic malcoaptation of the aortic valve leaflets (yellow arrow). (**F**) Three-dimensional CT reconstruction of the heart and thoracic aorta. Ascending thoracic aortic aneurysm. (**G**) CT reconstruction of the aortoiliofemoral vasculature for TAVR planning. Assessment of vascular anatomy and minimal luminal diameters. (**H**) Three-dimensional CT reconstruction for Ross procedure planning. Three-dimensional assessment of the aortic and pulmonary roots and the RVOT. (**I**) Multiplanar CT reconstruction of the RVOT. Cross-sectional measurement of the subpulmonic muscular cuff, 3–5 mm below the pulmonary annulus, including maximal and minimal diameters, area, and perimeter. Abbreviations: CT, computed tomography; LC, left coronary cusp; LVOT, left ventricular outflow tract; RC, right coronary cusp; RVOT, right ventricular outflow tract; TAVR, transcatheter aortic valve replacement.

CCT allows identification of AV morphology including identification of the number of cusps, the presence of raphe, and leaflet thickening or prolapse. CCT offers “en face” views of the AV from both the aortic or ventricular aspect, aiding in the visualization of restricted cusp motion that may contribute to AR and could be less well visualized by other imaging modalities [79] (Figure 7A–E).

An important application of CCT is in the precise measurement of aortic root dimensions, including the annulus, the sinuses of Valsalva, the STJ, and the proximal ascending aorta (Figure 7F). These structures are often implicated in chronic AR, either as primary pathologies (e.g., annuloaortic ectasia, connective tissue disorders such as Marfan or Loeys–Dietz syndrome) or secondary to volume overload. CT’s ability to measure these structures with high accuracy and reproducibility allows serial assessment and appropriate surgical planning, particularly when valve-sparing root replacement is considered or when determining the indication for concurrent aortic surgery. There is a class 1 recommendation to measure from the inner-to-inner edge of the aorta when using CCT or CMR imaging for aneurysm detection on cardiac-gated imaging, and outer-to-outer edge when aortic wall disease is present [80].

In addition to valve and root evaluation, CCT is instrumental in assessing the entire thoracic aorta for dilation, dissection, or coexisting atherosclerotic disease. Chronic severe AR can be associated with ascending aortic aneurysm, which may necessitate simultaneous surgical intervention, when dilated above specific threshold or showing rapid progression. Measurements of the aortic diameter should be performed with a three-dimensional approach using multi-planar reconstruction perpendicular to the long axis of the aorta. Automated or semi-automated aorta segmentation approaches with centerline scrolling are recommended and will be reproducible if imaging quality is adequate. This standardized approach ensures consistency across imaging modalities and facilitates accurate monitoring of aortic dimensions over time.

TTE is the preferred imaging modality for follow-up of patients with aortic root and proximal ascending aortic dilatation when these segments are adequately visualized and baseline dimensions have been confirmed by CCT or CMR. As mentioned above, echocardiographic measurements should be obtained in end-diastole using the leading-edge-to-leading-edge convention, which shows the best agreement with inner-to-inner CT/CMR measurements. If TTE and cross-sectional imaging measurements differ by more than 3 mm, follow-up should be based on CCT or CMR. CT or CMR is also preferred for dilatation involving the distal ascending aorta, aortic arch, or descending thoracic aorta [81].

### 5.2. Quantification of AR Severity

Blood flow cannot be directly measured via CCT; the anatomical regurgitant orifice area (AROA) can be measured via planimetry (Figure 7C). CCT can be played as a cine loop and offers dynamic information about leaflet coaptation and root motion (Figure 7E). Current prognostic data for chronic AR are based on echocardiography AR assessment or CMR regurgitant assessment. Comparative studies of CCT and echocardiography show wide variations in cutoffs for CCT ROA in differentiating moderate from severe AR, from 37 mm^2^ to 75 mm^2^ [82,83,84]. Some studies have found a more consistent correlation of CCT ROA with severe AR as defined by CMR, ranging from 23–27 mm^2^ [24]. Accordingly, CCT is not the modality of choice for AR quantification due to the lack of direct flow assessment and standardized AROA thresholds.

### 5.3. Evaluation of Left Ventricular Remodeling

Although mostly performed for other indications in AR patients, CCT can also provide additional measures of LV volumes and function, which are very important to assess the hemodynamic impact of AR, monitor progression and help set the indication for intervention. CCT-derived measurements of LV end-diastolic and end-systolic volumes have shown excellent agreement and correlation with CMR measurements [85].

### 5.4. CCT Assessment for TAVR Planning in Native Aortic Regurgitation

Transcatheter treatment of native aortic valve regurgitation (NAVR) has evolved from off-label use of conventional valves toward dedicated devices designed to address absent or mild leaflet calcification and annular and aortic root dilatation, which compromise anchoring and increase the risk of valve migration, embolization, and residual AR. In this context, advances in device design have improved anchoring mechanism and sealing capabilities, aiming to overcome these anatomical challenges. For example, the dedicated Trilogy valve and the J-valve incorporate three locators that capture the native leaflets between the locators and the valve frame, providing anchoring and sealing without relying on native valve calcification [86,87]. Clinical outcomes across successive studies illustrate this evolution. In the PANTHEON registry (*n* = 201), which predominantly used conventional platforms, technical success was 83.6%, and valve embolization or migration occurred in 12.4% [88]. More recently, the expanded ALIGN-AR study (*n* = 700) reported 95% technical success with Trilogy, with valve embolization in 1.3%; 30-day and 1-year mortality of 1.6% and 7.7%, respectively; and moderate residual AR in 0.5% of patients assessed at 30 days, with no severe cases [89]. The transfemoral J-Valve early feasibility study (*n* = 25) achieved successful implantation in 92%, with 4% mortality at both 30 days and 1 year and no moderate or severe residual AR through 1 year; the rate of embolization was not reported [86].

In this setting, CCT is central to anatomical screening and procedural planning, enabling assessment of valve morphology and leaflet calcification, systolic annular dimensions, LVOT and aortic root geometry, STJ dimensions, aortic angulation, coronary ostial heights, and ascending aortic disease. Evaluation of vascular access includes minimum luminal diameters, calcification, and tortuosity. Dedicated semiautomated structural heart software facilitates anatomical reconstruction and measurement extraction, although expert verification remains essential.

Anatomy in NAVR differs from that in calcific AS. Although measurements in pure NAVR patients are less challenging due to lack of calcification, there are distinct challenges with regard to device sizing, anchoring, and implantation. In a multicenter study by Gogia et al., 199 patients underwent CCT screening for the JenaValve Trilogy ALIGN-AR and ALIGN-AS studies, including 107 patients with severe native AR and 92 with severe AS. Compared with AS, patients with AR had larger mean annular areas (514 vs. 436 mm^2^), perimeters (81 vs. 75 mm), and maximum and minimum annular diameters (28 vs. 26 mm and 23 vs. 21 mm, respectively; all *p* < 0.0001). Sinus of Valsalva diameters were also larger in AR at the left coronary, right coronary, and noncoronary sinuses (37 vs. 32 mm, 35 vs. 30 mm, and 37 vs. 32 mm, respectively), as were maximum and minimum STJ diameters (35 vs. 29 mm and 33 vs. 27 mm) and maximum ascending aortic diameter (39 vs. 35 mm; all *p* < 0.0001). Mean annular angulation was greater in AR (53° vs. 48°; *p* = 0.0022), whereas left main and right coronary ostial heights did not differ significantly. On qualitative visual assessment, leaflet calcification was mild or absent in 86.9% of patients with AR, whereas 85% of patients with AS had severe calcification. Using an annular perimeter > 90 mm and annular angulation > 70° as anatomical exclusion criteria for the JenaValve Trilogy system, 15 patients with AR (14%) exceeded the perimeter limit, and six (6%) exceeded the angulation limit. Conversely, high-risk anatomy for coronary obstruction, defined as a sinus of Valsalva diameter < 30 mm combined with a coronary ostial height < 12 mm, was less frequent in AR than in AS for both coronary arteries [90].

In contrast, the transfemoral J-Valve early feasibility study allowed annular perimeters of 57–104 mm; 10 of 25 patients (40%) had annular perimeters > 90 mm, exceeding the upper eligibility limit for the aforementioned Trilogy device [89].

### 5.5. CCT Assessment for Ross Procedure Planning

The Ross procedure (replacement of the diseased aortic valve with the patient’s pulmonary autograft, followed by replacement of the pulmonary valve with a pulmonary allograft or another suitable valve prosthesis) represents an alternative to conventional prosthetic AVR in selected young or middle-aged patients [91]. The 2020 ACC/AHA guidelines state that, in patients younger than 50 years who prefer a bioprosthetic valve and have appropriate anatomy, AVR with a pulmonary autograft may be considered at a Comprehensive Valve Center (Class IIb, Level of Evidence B-NR) [28]. The 2025 ESC/EACTS guidelines recognize pulmonary autograft implantation as a potential alternative to prosthetic valve replacement in young individuals [13]. More recently, the EACTS Expert Consensus Statement identified aortic stenosis or mixed aortic valve disease with predominant stenosis, particularly in the presence of a non-dilated aortic annulus (below 27 mm), as the optimal indication for the Ross procedure. Nevertheless, patients with pure aortic regurgitation and a non-repairable aortic valve may also be suitable candidates when an appropriate autograft stabilization technique is used [92].

In this context, CCT has a central role in the preoperative assessment of coronary anatomy, aortic and pulmonary valve morphology, aortic–pulmonary annular mismatch, aortic and pulmonary root geometry, and the dimensions of the aortic root and ascending aorta (Figure 7H). As both right and left heart structures need evaluation, biventricular opacification is recommended, and specific contrast injection protocols (triphasic—two-phase contrast injection followed by a saline flush) should be applied [93].

Given the increased prevalence of anomalous coronary origins among patients with congenital aortic valve disease, coronary anatomy should be assessed by CTA (or angiography) in all Ross candidates [94]. CCT evaluation should include the coronary origin and proximal course, including the presence, location, and length of any intramural segment, and its relationship to the aortic root, pulmonary root, and right ventricular infundibulum. Retroaortic and interarterial courses may increase procedural complexity and represent relative contraindications, whereas a coronary artery crossing the right ventricular infundibulum (e.g., the right coronary artery originating from the LAD) is considered an absolute contraindication because of the risk of injury during pulmonary autograft harvesting [92].

Aortic valve morphology should be assessed, although bicuspid and unicuspid valve morphology have not been associated with an increased risk of autograft failure. Pulmonary valve morphology should also be evaluated, including cusp number and commissural orientation. Valve function and cusp fenestrations require complementary echocardiographic assessment and intraoperative inspection, respectively. Both annuli should be measured in their basal ring (BR) planes to identify potential mismatch. An aortic annulus more than 2 mm larger than the pulmonary annulus may increase the risk of autograft failure unless annular reduction or stabilization is performed, whereas a larger pulmonary annulus usually does not require additional correction [92]. Given the potentially elliptical geometry of both annuli, perimeter-derived diameters (perimeter/π) may provide a more accurate parameter for aortic–pulmonary size matching than linear diameters alone. Additional left-sided assessment should include LVOT dimensions and the presence, extent and location of LVOT calcification, as well as associated abnormalities, including subaortic membranes. Beyond pulmonary annular dimensions, CCT assessment should include measurements of the RVOT muscular cuff, 3–5 mm below the pulmonary annulus, as well as the pulmonary artery at the level of the bifurcation, including maximal and minimal diameters, average diameter, cross-sectional area, perimeter and perimeter-derived diameter (Figure 7I).

In a study by Jelenc et al., 50 coronary CTA examinations were evaluated to compare the in vivo dimensions and geometry of normal aortic and pulmonary roots and cusps. At end-diastole, the pulmonary root showed a distinct geometry, with a larger and more elliptical BR than the aortic root (mean diameter: 30.2 ± 3.0 vs. 24.6 ± 2.7 mm; *p* < 0.001), but smaller commissural and STJ dimensions. Accordingly, the STJ-to-basal ring ratio was lower in the pulmonary root (0.82 ± 0.09 vs. 1.14 ± 0.12; *p* < 0.001; mean difference, 0.32; 95% CI: 0.29–0.35). Cusp dimensions were generally similar between valves: estimated free-margin length and commissural height were identical, while pulmonary cusp geometric height and insertion length were only 0.8 and 1.6 mm greater, respectively. In contrast, pulmonary cusp effective height was significantly lower (5.9 ± 1.6 vs. 8.4 ± 1.2 mm; *p* < 0.001). Aortic and pulmonary root and cusp dimensions were strongly correlated, except for effective height [95]. These measurements (BR diameter, STJ diameter, cusp geometric height, free-margin length, insertion length and commissural height) should be evaluated during pre-procedural planning to anticipate the geometric remodeling required to achieve an aortic root-like autograft configuration and potentially reduce the risk of autograft regurgitation or failure.

Based on this comprehensive evaluation, different surgical techniques can be applied to achieve and maintain the optimal autograft geometry, including tailored free-standing root replacement with annular and/or STJ stabilization, autologous inclusion, and prosthetic inclusion techniques [92].

### 5.6. Limitations and Emerging CCT Techniques

#### 5.6.1. Technical Limitations

For anatomical assessment and procedural planning, CCT involves exposure to ionizing radiation, particularly relevant in younger patients, such as those undergoing Ross procedure planning, and in patients requiring repeated imaging for aortopathy surveillance [96]. The use of iodinated contrast media requires individualized risk assessment, particularly in patients with acute kidney injury or severe renal impairment [97]. Arrhythmias and respiratory or patient motion may compromise image quality. Multiphase ECG-synchronized acquisition allows selection of the least motion-affected cardiac phase, although it does not eliminate motion artifacts and may increase radiation exposure. Measurement accuracy depends on appropriate cardiac phase selection, correct identification of anatomical landmarks, and multiplanar alignment; semiautomated measurements therefore require expert verification, particularly in challenging anatomies [98].

#### 5.6.2. Emerging Techniques: Photon-Counting Detector CT and PET/CT Myocardial Perfusion

Photon-counting detector CT (PCD-CT) is an emerging technology that uses semiconductor detectors to count individual X-ray photons and measure their energy, providing intrinsic spectral information. Unlike conventional energy-integrating detector CT, low-energy monoenergetic reconstructions enhance iodine conspicuity, allowing improved vascular opacification with reduced contrast volumes. Recent experience in structural heart intervention planning has demonstrated the feasibility of using reduced contrast volumes of 30–50 mL of iodinated contrast, potentially benefiting patients with impaired renal function [99].

In selected patients with new or worsening AR, particularly in the setting of a prosthetic aortic valve and suspected infective endocarditis (PVE) based on clinical or laboratory findings, echocardiographic assessment is frequently limited by acoustic shadowing and prosthesis-related artifacts. In such cases, [^18^F]FDG PET/CT can provide valuable complementary information by identifying focal or heterogeneous paravalvular tracer uptake that confirms active infection and helps localize perivalvular complications such as aortic root abscess and pseudoaneurysm [100,101,102,103]. For PVE, its diagnostic performance is high—pooled sensitivity of approximately 86–93% and specificity of approximately 84–90%—and incorporating abnormal cardiac FDG uptake as a major criterion reclassifies a large proportion of “possible” cases to “definite,” raising the sensitivity of the Duke criteria from roughly 52–70% up to 91–97% [101,104,105]. Because the standardized imaging distinguishes anatomic from metabolic lesions, combining FDG PET with ECG-gated cardiac CT angiography (PET/CCT) in a single session, ideally with image coregistration, further improves detection of paravalvular abscesses and pseudoaneurysms by pairing the high sensitivity of PET for infection with the spatial resolution of CTA for structural lesions of the aortic root [106]. Important caveats include markedly lower sensitivity in native valve endocarditis (as low as ~22%), risk of false negatives with low systemic inflammation (CRP < 40 mg/L) or after prolonged antibiotics, risk of false positives from sterile postoperative inflammation, and the mandatory pre-scan dietary preparation to suppress physiologic myocardial glucose uptake [100,104,107]. The regurgitation severity and hemodynamic consequences themselves remain best characterized by echocardiography, with PET/CT serving to establish the infective etiology and map the perivalvular extent that guides surgical planning [100,103].

Quantitative myocardial perfusion may be an early biomarker of LV maladaptation as coronary reserve drops with aortic regurgitation. A Doppler study in dogs demonstrated that intracoronary flow reserve decreased from 3.51 at baseline to 1.20 with severe AR [108]. In a human study, 8 patients with chronic AR and exertional angina and without coronary stenosis were compared to 8 controls. Coronary flow reserve was markedly reduced in AR (1.67 ± 0.40 vs. 4.03 ± 0.52, *p* < 0.001), driven largely by increased resting coronary blood flow with limited further vasodilator augmentation [109]. Although these studies were not based on cardiac PET, PET is currently one of the preferred noninvasive modalities for quantifying myocardial flow reserve, alongside quantitative stress perfusion CMR. These studies provide mechanistic evidence for a possible cardiac PET biomarker requiring further study in the future.

## 6. Multimodal Imaging in Acute Aortic Regurgitation

Acute severe AR differs substantially from chronic disease due to the absence of compensatory chamber dilation. Sudden retrograde volume delivery in a non-compliant LV results in an abrupt elevation in end-diastolic pressure that rapidly approaches aortic diastolic pressure. Consequently, forward cardiac output drops precipitously, frequently resulting in flash pulmonary edema or cardiogenic shock. Because LV remains normal, standard chronic remodeling criteria cannot be applied. The LV typically exhibits normal or hyperdynamic systolic function. Severe elevation of ventricular diastolic pressure causes premature mitral valve closure before the onset of the QRS complex, visible on M-mode and PW Doppler tracings. The rapid equilibration between aortic and LV pressures leads to a steep CW Doppler deceleration slope and remarkably truncated PHT, frequently <200 ms. The imaging algorithm is dictated by the underlying structural emergency causing acute valvular decompensation [10]. In case of suspected infective endocarditis, urgent TTE should be followed by TEE if images are non-diagnostic. 3D TEE may help characterize cusp perforations, ruptured fenestrations, and root abscesses with high sensitivity. If acute aortic syndrome is suspected, urgent CCT is required. It provides cross-sectional imaging of the aortic root and sinotubular geometry, and coronary arteries when emergency surgical reconstruction or replacement is planned. TTE in this clinical scenario demonstrates a pooled sensitivity of 58% (95% CI: 38–76%) and specificity of 94% (95% CI: 89–97%) for type A dissection [110]. In iatrogenic procedural failure, intraprocedural TEE remains the preferred modality to localize paravalvular regurgitation, valve migration, or mechanical impingement following surgical or transcatheter interventions.

## 7. Multimodality Imaging for Post-Treatment

Beyond the periprocedural period, which is outside the scope of this review, longitudinal echocardiographic surveillance is essential to assess residual or recurrent AR following surgical aortic valve repair, paravalvular leak (PVL) or transvalvular regurgitation following TAVR, and LV reverse remodeling after intervention. In the expanded ALIGN-AR cohort, moderate or greater residual AR was observed in 1% of patients at 1 year, while the transfemoral J-Valve early feasibility study reported no moderate or severe residual AR among 22 patients evaluated at 1 year [89]. Echocardiographic assessment of PVL includes an integrated approach incorporating color Doppler evaluation of jet location and circumferential extent, VC measurements and diastolic flow reversal in the descending aorta, with 3D TEE providing additional anatomical characterization when needed [111].

CCT provides complementary anatomical assessment in suspected prosthetic valve dysfunction, including hypoattenuated leaflet thickening (HALT) and reduced leaflet motion. Although HALT has been reported in approximately 10–30% of aortic bioprostheses, evidence is predominantly derived from patients treated for aortic stenosis, and its incidence following TAVR for isolated NAVR remains insufficiently characterized.

Following aortic valve repair involving root or ascending aortic reconstruction, CCT provides complementary anatomical assessment of graft integrity, anastomotic complications, pseudoaneurysms, and residual aortic dilation. Subsequent surveillance should be individualized according to the surgical procedure, underlying aortopathy, and postoperative findings [112].

Following the Ross procedure, lifelong echocardiographic surveillance is recommended to assess pulmonary autograft function, neo-aortic root dilation, and pulmonary homograft dysfunction, with CCT or CMR providing complementary anatomical and functional assessment when indicated [92].

CMR provides complementary assessment of ventricular volumes and function, myocardial tissue characterization (including LGE and ECV), and quantification of residual AR when echocardiographic assessment is inconclusive.

## 8. Conclusions

AR is a common valvular disease associated with adverse outcomes when significant disease is not diagnosed or intervention is delayed. The diagnostic approach should reflect its clinical presentation: acute AR requires prompt identification of the underlying cause and hemodynamic consequences, whereas chronic AR requires accurate assessment of regurgitation severity, ventricular remodeling, and disease progression to guide surveillance and timely intervention.

Echocardiography remains the cornerstone and first-line imaging modality. Complementary or adjudicative imaging should be selected according to the clinical question, including clarification of valve anatomy and mechanism, quantification of regurgitation, characterization of ventricular remodeling and myocardial fibrosis, or procedural planning. Effective integration requires an understanding of the strengths of each modality and its limitations and potential sources of error. Severity/prognostic thresholds should be specific to each modality rather than assumed to be interchangeable, given differences in methodology. As CMR assumes an increasingly prominent role in valvular heart disease, further validation studies are needed to move from significant AR thresholds based on observations of adverse outcomes to prospectively studied treatment thresholds based on improved outcomes from surgical or transcatheter interventions. Advances in image acquisition and post-processing, including photon-counting detector CT, evolving CMR techniques, and advanced software-assisted image analysis, may improve image quality, measurement reproducibility and workflow efficiency. These developments may further refine structural assessment and procedural planning, although their clinical application requires appropriate validation and expert interpretation.

Ultimately, AR assessment should be both multimodal and comprehensive. An integrated approach should consider whether AR is acute or chronic, symptoms, the impact on cardiac chambers, progression over time, frailty, and patient preferences. Whenever possible, patients should be followed at specialized centers with dedicated heart valve clinics. When such care is unavailable, timely referral is essential to avoid delays in diagnosis and appropriate intervention.

## Figures and Tables

**Table 1:** Strengths, limitations, and clinical indications for modalities used in severe aortic regurgitation evaluation and treatment planning.

Modality	Key Strengths	Limitations	Clinical Indications
TTE	Widely accessible Low cost Incorporates CW Doppler, PW flow reversal, and Color Doppler to characterize flow dynamics across the cardiac cycle	Dependent on acoustic windows Poor accuracy in eccentric jets PISA and VC calculations assume circular orifice and planar flow convergence, frequently fail in eccentric or multiple jets PHT and descending aorta flow reversal depend heavily on chamber compliance, systemic vascular resistance, and heart rate	First-line diagnostic modality Severity grading Routine surveillance
TEE	Superior anatomical resolution for leaflet prolapses and perforations, especially with 3D acquisition Intraprocedural guidance for transcatheter and surgical procedures	Semi-invasive procedure Requires esophageal access Position-dependent imaging windows (far field degrades)	Inadequate TTE windows Infective endocarditis Native valve repair or valve-sparing root procedure feasibility Intraprocedural guidance
3D echocardiography	Real-time en face visualization of the valve Direct planimetry of VC area (3D VCA) eliminating geometric shape assumptions Quantification of eccentric jets	Reduced temporal resolution Stitching artifacts in multibeat acquisition due to respiratory motion or arrhythmias Dropout artifacts due to calcifications or presence of prostheses	Repair-oriented surgical planning Transcatheter sizing Precise evaluation of paravalvular leaks
CMR	Reference standard for noninvasive quantification of regurgitant fraction using phase-contrast flow and 4D flow mapping and volume evaluation Detects myocardial fibrosis via late gadolinium enhancement and extracellular volume calculation	Limited availability of experts Substantial acquisition time and repeated breath-holding Susceptibility to arrhythmic gating errors Myocardial tissue characterization using LGE and ECV requires gadolinium-based contrast administration, warranting caution in patients with acute renal dysfunction	Class 1 recommendation when echo findings are discordant or inconclusive, in eccentric regurgitant jets, or in suboptimal acoustic windows Precise LV volumetry and tissue characterization enabling insight into irreversible myocardial injury
CCT	High-resolution visualization of aortic root and aorta dimensions, cusp morphology and calcifications, annular geometry Coronary assessment	Ionizing radiation, iodinated contrast nephrotoxicity risk Static flow evaluation Lacks direct flow quantification	Pre-procedural planning for transcatheter approaches Assessing feasibility of valve repair or replacement
[^18^F] FDG PET/CT	Direct evaluation of active vascular and valvular inflammation by evaluation of 18F-FDG accumulation in regions of macrophage infiltration	Ionizing radiation Limited spatial resolution	Suspected prosthetic valve endocarditis or infected composite aortic grafts

Abbreviations: CCT, Cardiac Computed Tomography; CMR, Cardiac Magnetic Resonance; 18F-FDG, 18F-fluorodeoxyglucose; TTE, transthoracic echocardiography; TEE, transesophageal echocardiography.

**Table 2:** Multimodality imaging parameters and reported cut-offs for severe aortic regurgitation [9,10,13,24,25].

Echocardiography	
Qualitative	Holodiastolic flow reversal in descending aorta (EDV ≥ 20 cm/s) Diastolic flow reversal in abdominal aorta
Semi-quantitative	VC width > 6 mm PHT < 200 ms Large central jet ≥ 65% of LVOT diameter CSA jet/CSA LVOT ≥ 60%
Quantitative	EROA ≥ 30 mm^2^ RVol ≥ 60 mL/beat RF ≥ 50%
CMR	
Quantitative †	RF ≥ 50% RVol ≥ 60 mL/beat
CCT	
Quantitative *	AROA 23–27 mm^2^ (CMR-defined severe AR)

Abbreviations: CMR, cardiovascular magnetic resonance; CSA, cross-sectional area; EDV, end-diastolic velocity; EROA, effective regurgitant orifice area; LVOT, left ventricular outflow tract; PHT, pressure half-time; RF, regurgitant fraction; RVol, regurgitant volume; VC, vena contracta; CCT, cardiac computed tomography angiography; AROA, anatomical regurgitant orifice area. † CMR severity thresholds are derived from echocardiographic criteria. * Published CCT AROA thresholds vary according to the reference standard; thresholds of 37–75 mm^2^ have been reported versus echocardiography and 23–27 mm^2^ versus CMR.

**Table 3:** Comparison of surgical and interventional indications for chronic aortic regurgitation between 2020 ACC/AHA and 2025 ESC guidelines [13,28].

	2020 ACC/AHA Guidelines	2025 ESC/EACTS Guidelines
Symptomatic Severe AR	Class I recommendation for surgery regardless of LV function	Class I recommendation for surgery regardless of LV function
Asymptomatic Severe AR	Class I recommendation for LVEF ≤ 55% Class 2a in patients with LVEF > 55% and LVESD > 50 mm or LVESDi > 25 mm/m^2^ Class 2b in patients with low surgical risk with progressive decline in LVEF on at least 3 studies < LVEF 55–60% or a progressive increase in LVEDD > 65 mm	Class I recommendation for LVESD > 50 mm or LVESDi > 25 mm/m^2^ (especially in patients with small BSA < 1.68 m^2^) or resting LVEF ≤ 50% Class II b recommendation for LVESDi > 22 mm/m^2^ or LVESVi > 45 mL/m^2^ (especially in patients with small BSA < 1.68 m^2^) or resting LVEF ≤ 55% if surgical risk is low
Aortic Valve Repair	Valve-sparing strategy may be possible in selected patients with favorable valve anatomy undergoing surgical replacement of the aortic sinuses and/or ascending aorta 2b recommendation in patients with BAV at experienced centers	Class IIa recommendation when durable reconstructive results are anticipated
Transcatheter Therapy (TAVR)	Class III for isolated severe AR in operable patients	Class II b consideration in selected inoperable native AR
Concomitant Cardiac Surgery	Class I recommendation in patients with severe AR undergoing cardiac surgery for other indications Class IIa recommendation for patients with moderate AR undergoing cardiac or aortic surgery for other indications	Class I recommendation in symptomatic or asymptomatic severe AR undergoing CABG or ascending aorta surgery

Abbreviations: AR, aortic regurgitation; BSA, body surface area; BAV, bicuspid aortic valve; CABG, coronary artery bypass grafting; LV, left ventricle; LVEF, left ventricular ejection fraction; LVEDD, left ventricular end-diastolic diameter; LVESD, left ventricular end-systolic diameter; LVESDi, left ventricular end-systolic diameter index; LVESVi, left ventricular end-systolic volume index.

**Table 4:** CMR-derived RF and VRol thresholds associated with outcomes in chronic AR.

Study	Population	Threshold Purpose	RF/RVol Cutoff	Endpoint	Main Limitation
Myerson et al., 2012 [52]	*n* = 113; asymptomatic; moderate-to-severe AR by TTE	Prognosis	RF > 33%; RVol > 42 mL	Progression to symptoms or surgical indications (AUC 0.93 and 0.96, respectively)	Limited sample size; exercise testing not reported; potential decision bias due to unblinded CMR results in 3/4 centers; no external validation
Hashimoto et al., 2022 [53]	*n* = 178; ≥moderate AR; 158 NYHA I–II	Prognosis	RF ≥ 32%	Independently predicted death, HF hospitalization, or worsening NYHA class beyond LVESVi ≥ 45 mL/m^2^ (adjusted HR 3.18; 95% CI 1.60–6.34; AUC 0.70).	Retrospective; moderate discrimination (AUC 0.70); no external validation
Vejpongsa et al., 2022 [54]	*n* = 390; isolated AR	Prediction of AVR	RF > 35%; RVol > 38 mL	Predicted subsequent AVR (AUC 0.94 and 0.95, respectively)	Retrospective; baseline symptoms not reported; potential referral/decision bias
Malahfji et al., 2023 [55]	*n* = 458; asymptomatic; ≥moderate AR	Prognosis	RF > 43%; RVol ≥ 47 mL	Symptoms, LVEF < 50%, AVR referral for LV dilation, or death (AUC 0.63 and 0.64, respectively).	Modest discrimination; composite endpoint includes AVR referral decisions.

Abbreviations: AR, aortic regurgitation; AUC, area under the receiver operating characteristic curve; AVR, aortic valve replacement; CMR, cardiovascular magnetic resonance; HR, hazard ratio; LV, left ventricular; NYHA, New York Heart Association functional class; RF, regurgitant fraction; RVol, regurgitant volume; TTE, transthoracic echocardiography.

## Data Availability

No new data were created or analyzed in this study. Data sharing is not applicable to this article.

## Abbreviations

The following abbreviations are used in this manuscript:

2D	Two-dimensional
3D	Three-dimensional
4D flow CMR	Time resolved, 3D phase-contrast imaging
AR	Aortic regurgitation
AROA	Anatomical regurgitant orifice area
AV	Aortic valve
AVR	Aortic valve replacement
BAV	Bicuspid aortic valve
BSA	Body Surface Area
BR	Basal ring
CMR	Cardiac Magnetic Resonance
CCT	Cardiac Computed Tomography
CW	Continuous-wave Doppler
ECV	Extracellular volume
EROA	Effective regurgitant orifice area
gH	Geometric height
GLS	Global longitudinal strain
HF	Heart failure
iECV	Indexed extracellular volume
LA	Left atrium
LCC	Left coronary cusp
LV	Left ventricle
LVEF	Left ventricular ejection fraction
LVEDVi	Left ventricular end-diastolic volume index
LVESD	Left ventricular end-systolic diameter
LVESDi	Left ventricular end-systolic diameter index
LVESVi	Left ventricular end-systolic volume index
LVOT	Left ventricular outflow tract
MV	Mitral Valve
MR	Mitral Regurgitation
NCC	Non-coronary cusp
PLAX	Parasternal long-axis view
PHT	Pressure-half time
PISA	Proximal isovelocity surface area
PW	Pulsed-wave Doppler
PVE	Prosthetic valve endocarditis
RCC	Right coronary cusp
RF	Regurgitant Fraction
RVol	Regurgitant volume
SAX	Short-axis view
STJ	Sinotubular junction
TAV	Tricuspid Valve
TVR	Transcatheter Aortic Valve Replacement
TEE	Transesophageal echocardiography
TG	Transgastric views
TTE	Transthoracic echocardiography
VC	Vena contracta
VSARR	Valve-sparing aortic root replacement
